# Therapeutic effects of Silexan on somatic symptoms and physical health in patients with anxiety disorders: A meta‐analysis

**DOI:** 10.1002/brb3.1997

**Published:** 2021-02-27

**Authors:** Roland von Känel, Siegfried Kasper, Guido Bondolfi, Edith Holsboer‐Trachsler, Josef Hättenschwiler, Martin Hatzinger, Christian Imboden, Ellen Heitlinger, Erich Seifritz

**Affiliations:** ^1^ Department of Consultation‐Liaison‐Psychiatry and Psychosomatic Medicine University Hospital Zurich University of Zurich Zurich Switzerland; ^2^ Department of Psychiatry and Psychotherapy Medical University of Vienna Vienna Austria; ^3^ Department of Psychiatry University Medical Centre University of Geneva Geneva Switzerland; ^4^ Gesundheitszentrum St. Johann University of Basel Basel Switzerland; ^5^ Centre for Anxiety and Depression Treatment Zurich Zurich Switzerland; ^6^ Psychiatric Services Solothurn and University of Basel Solothurn Switzerland; ^7^ Private Clinic Wyss Münchenbuchsee Switzerland; ^8^ H+O communications Ltd. Zurich Switzerland; ^9^ Department of Psychiatry, Psychotherapy and Psychosomatics Psychiatric Hospital University of Zurich Zurich Switzerland

**Keywords:** anxiety disorders, anxiolytics, herbal therapy, meta‐analysis, quality of life, somatic symptoms

## Abstract

A meta‐analysis was performed to examine therapeutic effects of Silexan on somatic symptoms, including insomnia/fatigue, and physical health in patients with anxiety disorders. Five randomized, placebo‐controlled trials were included in this analysis: The efficacy of Silexan (80 mg/day) was investigated in patients with subthreshold anxiety disorders (three trials) and in patients with generalized anxiety disorder (two trials). Silexan was superior to placebo in terms of the mean change from baseline in the Hamilton Anxiety Rating Scale (HAMA) subscore somatic anxiety at week 10 with a standardized mean difference of −0.31 [95% Cl: −0.52 to −0.10, *p* = .004]. Treatment effects of silexan on somatic anxiety were independent of gender and age. Statistically significant differences were also shown for single HAMA items somatic muscular, cardiovascular, respiratory, and genitourinary symptoms, indicating clinical relevance with small to medium effects of Silexan. Similar clinically meaningful effects of Silexan on SF‐36 physical health, including reduced bodily pain and improved general health, and on insomnia complaints and fatigue, were demonstrated. In this meta‐analysis including all placebo‐controlled clinical trials in patients with anxiety disorders to date, statistically significant and clinically meaningful advantages of Silexan over placebo treatment were found in improving somatic symptoms and physical health.

## Abstract 2

In this meta‐analysis including all placebo‐controlled clinical trials in patients with anxiety disorders to date, statistically significant and clinically meaningful advantages of Silexan over placebo treatment were found in improving somatic symptoms and physical health.

## 1 INTRODUCTION

Anxiety disorders are recognized among the most frequent categories of mental illnesses worldwide (Kessler et al., 2007), with an estimated mean age of onset of 21.3 years (de Lijster et al., 2017). According to large epidemiological surveys, up to 33.7% of the population are affected by an anxiety disorder during their lifetime (Bandelow & Michaelis, 2015). Generalized anxiety disorder (GAD) and subthreshold anxiety (i.e., defined in patients with clear impairment that do not meet the required number of symptoms of threshold definitions) are highly prevalent and impairing conditions among both adolescents and adults (Baldwin et al., 2011; Burstein et al., 2014; Haller et al., 2014; Siegel & Dickstein, 2011). In a primary healthcare setting, the prevalence of GAD in adult patients has been reported as 4.1%–6.0% among men, and 3.7%–7.1% among women (Munk‐Jorgensen et al., 2006). Prevalence data from a community mental health survey reported that threshold and subthreshold anxiety affect similar percentages of adolescents aged 15 years or older (2.6% versus. 2.3%, respectively) (Gilmour, 2016). GAD and subthreshold anxiety share the same common elements of disproportionate and debilitating worry characterizing all anxiety disorders (Burstein et al., 2014). According to the Diagnostic and Statistical Manual of Mental Disorders, fifth edition (DSM‐5), somatic symptoms belong to the definition criteria of GAD, including restlessness, increased fatigability, muscle tension, and sleep disturbance (American Psychiatric Association (2013)). These symptoms may be accompanied by autonomic hyperarousal, that is, sweating, dizziness, and shortness of breath (Gelenberg, 2000). If untreated, anxiety symptoms persist and are associated with significant impairments in daily functioning, poor quality of life, and a huge economic burden owing to lost work productivity and high healthcare utilization costs (Greenberg et al., 1999; Hoffman et al., 2008).

Typically, patients with GAD present with mostly somatic complaints (Crawford et al., 1994) that vary between individuals. Somatic symptoms tend to be prolonged in patients with anxiety disorders. However, many anti‐anxiety drugs such as benzodiazepines, other gamma‐aminobutyric acid (GABA)‐facilitatory drugs, for example, clomethiazole, azapirones, for example, buspirone, and antidepressants are contraindicated for treatment because of side effects and/or the risk of dependence associated with chronic use (Morgan & Tyrer, 1994).

The anxiolytic efficacy of the orally administered lavender oil preparation Silexan has been investigated in GAD and other anxiety disorders (Kasper et al., 2010, 2016; Kasper, Gastpar, Muller, et al., ). In a meta‐analysis of three randomized, placebo‐controlled trials (Kasper et al., 2010, 2015, 2016), Silexan demonstrated superiority to placebo in reducing somatic symptoms and improving physical health in patients with subthreshold anxiety (Möller et al., 2019). Secondary to its anxiolytic effect, Silexan had a beneficial effect on sleep, often accompanied by fatigue, and it also improved patients' health‐related quality of life (HRQoL) (Möller et al., 2019). Five placebo‐controlled clinical trials investigating the efficacy of Silexan (80 mg/day) in patients with anxiety disorders have been performed to date (Kasper et al., 2010, 2015, 2016, 2017; Kasper, Gastpar, Müller, et al., 2014). We conducted a meta‐analysis of all five clinical trials to further elucidate the therapeutic effects of Silexan with respect to somatic complaints in patients with anxiety. Besides including two additional studies, the novelty of the current versus a previous meta‐analysis (Möller et al., 2019) is a detailed investigation of individual anxiety‐related somatic symptoms, including pain, insomnia complaints along with fatigue, and physical health (Eliasen et al., 2016).

## 2 METHODS

### 2.1 Clinical trials included

In the meta‐analysis, placebo‐controlled clinical trials investigating the efficacy of Silexan in patients with anxiety disorders were included. These trials are Kasper et al., 2010 (A), Kasper et al., 2015 (B), Kasper et al., 2017 (C), Kasper, Gastpar, Muller, et al.,  (D) and Kasper et al., 2016 (E). Since all trials included a group of patients receiving a dosage of 80 mg/day Silexan, and no other dosage was investigated in more than one trial, the efficacy of 80 mg/day Silexan was compared to placebo in the meta‐analysis. Individual patient data are used for the meta‐analysis. To identify any additional randomized placebo‐controlled clinical trials conducted with 80 mg Silexan in patients with anxiety disorders, one author performed literature searches by using the PubMed database. Search terms included “Silexan” in combination with “anxiety disorder” before 30 April 2019 (no further restrictions applied). The searches did not reveal any additional randomized placebo‐controlled clinical trial in patients with anxiety disorders.

### 2.2 Bias assessment

Bias assessment on the study level was performed using the Cochrane Collaboration's tool for assessing risk of bias (Higgins et al., 2011). Assessments were based upon the applicable publications, the patient raw data, and on the original protocols, and the full integrated study reports made available to the authors and to the assessor.

### 2.3 Outcome measures

The analysis of treatment effects was based on the full analysis sets (FAS) of the included clinical trials. Missing values were replaced by last observation carried forward (LOCF) as performed for the original analysis of the trials. The trial populations were characterized by the number of randomized patients, number of dropouts, sample size of the FAS, portion of female patients, and mean age of the patients. The Hamilton Anxiety Rating Scale (HAMA; Hamilton, 1959) is a measure for changes in severity of anxiety symptoms. The HAMA is a 14‐item clinician‐administered rating scale that measures the severity of anxiety based on the frequency and impairment of symptoms during the past week. Each item is rated on a five‐point scale from 0 (not present) to 4 (very severe). Higher scores indicate a greater degree of symptom severity. The primary outcome measure for this meta‐analysis was the sum of HAMA items 7–13, known as the somatic anxiety subscore, consisting of the following items: (item 7) somatic muscular (pains and aches, twitching, stiffness, myoclonic jerks, grinding of teeth, unsteady voice, increased muscular tone); (item 8) somatic sensory (tinnitus, blurring of vision, hot and cold flushes, feelings of weakness, pricking sensation); (item 9) cardiovascular (tachycardia, palpitations, pain in chest, throbbing of vessels, fainting feelings, sighing, dyspnoea); (item 10) respiratory (pressure or constriction in chest, choking feelings, sighing, dyspnoea); (item 11) gastrointestinal (difficulty in swallowing, wind, abdominal pain, burning sensations, abdominal fullness, nausea, vomiting, bowel sounds, looseness of bowels, loss of weight, constipation); (item 12) genitourinary (frequency of micturition, urgency of micturition, amenorrhea, menorrhagia, development of frigidity, premature ejaculation, loss of libido, impotence); and (item 13) autonomic symptoms (dry mouth, flushing, pallor, tendency to sweat, giddiness, tension headache, raising of hair). Additional single outcome measures describing somatic complaints included the following: somatic muscular (HAMA item 7); somatic sensory (HAMA item 8); cardiovascular symptoms (HAMA item 9); respiratory symptoms (HAMA item 10); gastrointestinal symptoms (HAMA item 11); genitourinary symptoms (HAMA item 12); autonomic symptoms (HAMA item 13); and, in all trials, except Kasper et al. (2015), the Short Form 36 (SF‐36). Of the latter, the subscore physical health (summary measure) with the 4 scaled components physical functioning, role‐physical, bodily pain, and general health were analyzed indicating physical HRQoL.

Two further outcomes describing sleep quality were investigated: insomnia (HAMA item 4; difficulty in falling asleep, broken sleep, unsatisfying sleep and fatigue on waking, dreams, nightmares, night terrors) and SF‐36 vitality as a measure of fatigue (Brown et al., 2011).

These subscales have been widely used in GAD research, and the symptoms within these subscale domains have previously been shown to be differentially sensitive to various treatment effects (Feighner & Cohn, 1989; Meoni et al., 2001; Pollack et al., 2001; Rickels et al., 1993; Rickelset al., 1982). In all studies, assessments were performed prior to treatment (baseline) and at week 10.

Treatment effects related to somatic symptoms by gender and age (<60 years and ≥ 60 years) were also analyzed.

### 2.4 Statistical methods

The meta‐analysis was planned after all trials had been conducted, and the methods including the choice of the outcomes were specified before the meta‐analysis was performed. Descriptive statistics (sample size, means and standard deviation (*SD*) for continuous data) were calculated for outcomes in the single trials (FAS) and for the pooled data set. Descriptive statistics of baseline values were also calculated. Treatment effects of Silexan within the single trials were compared to placebo using analysis of covariance models with factors for “treatment group” and the baseline value of the outcome variable as covariate. The results for the single trials were calculated using SAS 9.4, Windows 7 professional. The combination of the results (meta‐analysis) was performed using R package meta (version 4.3–2, function metacont). For each outcome variable, a random effects meta‐analysis was performed. The inverse variance weighting was used for combining the results of the single trials and the DerSimonian‐Laird method was used. Standardized mean differences (SMD) were calculated in order to use Cohen's categories for classifying the magnitude of the treatment effects in terms of clinical meaning. According to Cohen (1988) (Cohen J, 1988), an effect size of d = 0.20/ 0.50/ 0.80 describes a small/ medium/ large effect, respectively.

Treatment effects on somatic anxiety quantified by the change of the HAMA somatic anxiety subscore after 10 weeks of treatment were investigated separately for women and men. Treatment effects were compared within the subgroups following the strategy described above. The efficacy of Silexan 80 mg/day compared to placebo was investigated using mixed effects models assuming random effects within subgroups and a fixed effect across subgroups. The variance between trials was estimated separately for each subgroup. The p‐value of the Q test for heterogeneity was calculated (method 3) (Borenstein M et al., 2009) to compare results between the subgroups.

Results of the meta‐analyses are presented using forest plots (R, package meta; version 4.3–2, function forest). The p‐values of two‐sided tests are presented, a p‐value < 0.05 is considered statistically significant.

## 3 RESULTS

### 3.1 Characteristics of included studies

Three placebo‐controlled clinical trials investigating the efficacy of 80 mg/day Silexan in patients with subthreshold anxiety disorders (Kasper et al., 2010 (trial A) (Kasper et al., 2010), Kasper et al., 2015 (trial B) (Kasper et al., 2015), and Kasper et al., 2016 (trial C) (Kasper et al., 2016)), and two trials in patients with GAD (Kasper et al., 2017 (trial D) (Kasper et al., 2017) and Kasper, Gastpar, Muller, et al.,  (trial E) (Kasper, Gastpar, Müller, et al., 2014)) were analyzed. Patients with anxiety not otherwise specified (NOS) (Diagnostic and Statistical Manual of Mental Disorders, 4th Edition (DSM IV, 300.0) or International Classification of Diseases 10th Revision (ICD‐10, F41.9) in trial A, patients suffering from anxiety‐related restlessness and sleep disturbances (ICD‐10, R45.1) in trial B, and patients with mixed anxiety and depression (ICD‐10, F41.2) in trial C were included. From trial D, which compared three Silexan doses (10 mg, 40 mg, 80 mg) to placebo, and from the reference and placebo‐controlled trial E, only data of patients randomized to receive 80 mg/day Silexan or placebo were used in this meta‐analysis. In all five trials, the patients were treated for 10 weeks. Characteristics of the trial populations are shown in Table 1. Across all 5 studies, a total of 1,213 patients were randomized to receive either 80 mg/day Silexan or placebo. Of these, 1,172 patients could be analyzed for efficacy (FAS). Approximately 70% of the patients were women, and the mean age was approximately 46 years (Table 1).

**Table 1:** Characteristics of included trial populations

Clinical trial	Diagnosis	Treatment groups	Randomized *N*	Drop‐Outs^a^ *N* (%)	FAS *N*	Female (%)	Age Years, mean (*SD*)	Reference
A	Anxiety (NOS) (DSM IV 300.00, ICD−10, F41.9)	Silexan 80 mg	110	18 (16.4)	104	73.1%	45.6 (11.4)	(Kasper S et al., 2010)
		Placebo	111	14 (12.6)	108	76.9%	46.6 (11.3)	
B	Restlessness, agitation (ICD−10, R45.1)	Silexan 80 mg	86	12 (14.0%)	86	72.1%	48.0 (11.3)	(Kasper S et al., 2015)
		Placebo	84	10 (11.9%)	84	71.4%	46.9 (12.7)	
C	Mixed Anxiety and depression (ICD−10, F41.2)	Silexan 80 mg	160	15 (9.4%)	159	66.0%	47.7 (12.6)	(Kasper S et al., 2016)
		Placebo	158	13 (8.2%)	156	72.4%	47.9 (12.6)	
D^b^	GAD	Silexan 80 mg	118	11 (9.3%)	103	76.7%	43.3 (11.7)	(Kasper S et al., 2017)
		Placebo	113	8 (7.1%)	102	65.7%	45.5 (11.5)	
E^c^	GAD	Silexan 80 mg	136	17 (12.5%)	135	70.4%	45.7 (11.5)	(Kasper, Gastpar, Muller, et al., )
		Placebo	137	19 (13.9%)	135	73.3%	44.6 (12.3)	
Pooled	NR	Silexan 80 mg	610	73 (12.0%)	587	71.0%	46.1 (11.9)	NR
		Placebo	603	64 (10.6%)	585	72.1%	46.4 (12.2)	

Note

Abbreviations: DSM IV, Diagnostic and Statistical Manual of Mental Disorders, 4th Edition; FAS, full analysis set; GAD, general anxiety disorder; ICD‐10, International Classification of Diseases 10th Revision; NOS, not otherwise specified; NR, not relevant.

^a^ percent of randomized patients.

^b^ In trial D, additionally *n* = 117 / 113 patients were randomized to receive Silexan 40 mg / 10 mg, respectively.

^c^ In trial E, additionally *n* = 129 / 137 patients were randomized to receive Silexan 160 mg / paroxetine 20 mg, respectively.

### 3.2 Baseline values of analyzed outcomes

Baseline values of the HAMA subscore somatic anxiety, HAMA item 7–13 describing somatic symptoms, HAMA item 4 (insomnia), and the SF‐36 subscore physical health, and the SF‐36 components related to physical health and vitality (as a measure of fatigue) are shown in the appendix (Table A1 and Table A2).

At baseline, the mean values of the HAMA subscore somatic anxiety (between 10.3 and 12.0) indicated trial participants had at least mild‐to‐moderate severity of anxiety symptoms. The mean values of HAMA items 7 (somatic muscular), 8 (somatic sensory), 9 (cardiovascular symptoms), and 13 (autonomic symptoms) showed that patients suffered from symptoms of moderate intensity (mean values of pooled data between 1.7 and 2.0). Respiratory, gastrointestinal, and genitourinary symptoms of mild intensity were observed (mean values of pooled data between 1.2 and 1.4) at baseline. In addition, patients suffered from moderate to severe insomnia (HAMA item 4, pooled mean value of 2.7).

The patients had markedly reduced physical health at baseline, indicated by the corresponding SF‐36 subscore (mean values between 48.2 and 56.5). The baseline values were comparable between the treatment groups within the trials and in the pooled data set.

### 3.3 Treatment effects with respect to somatic complaints

**Figure 1:**
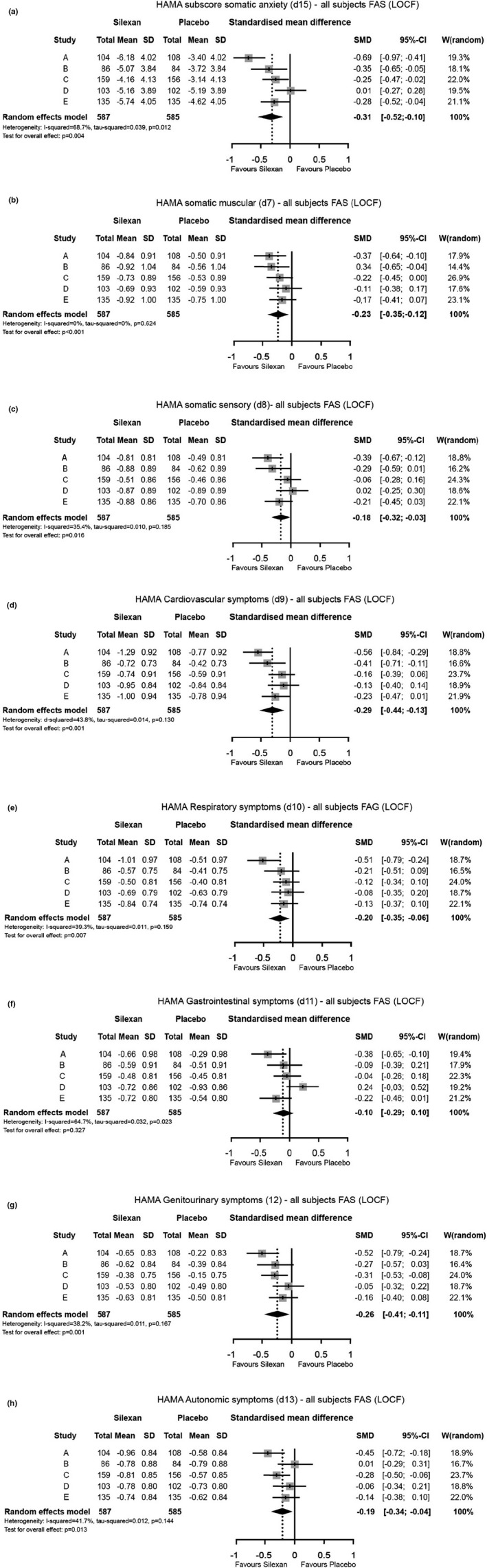
(a–h) Changes of HAMA somatic anxiety subscore and HAMA somatic complaints (item 7–13) between baseline and week 10

In 4 of the 5 trials analyzed, somatic anxiety improved more in patients treated with Silexan compared to the placebo groups; in 1 trial (trial D), there was no difference between treatment groups (Figure 1a). In the meta‐analyses, statistically significant and clinically meaningful differences between Silexan 80 mg/day and placebo were shown for the HAMA subscore somatic anxiety (*p* = .004) (Figure 1a) and for the single HAMA items 7 (somatic muscular) (Figure 1b), 9 (cardiovascular symptoms) (Figure 1d), 10 (respiratory symptoms) (Figure 1e), and 12 (genitourinary symptoms) (Figure 1g), describing somatic complaints. The SMD ranged between 0.20 (HAMA item 10, respiratory symptoms; Figure 1e) and 0.31 (HAMA subscore somatic anxiety; Figure 1a), indicating small to medium effects of Silexan on these somatic complaints. The strongest overall effects were observed for cardiovascular (*p* < .001) and genitourinary symptoms (*p* < .001). Treatment effects of Silexan on HAMA items 8 (somatic sensory) (Figure 1c) and 13 (autonomic effects) (Figure 1h) were also statistically significant, although in clinical terms, these effects were negligible (SMD < 0.20). In case of item 11 (gastrointestinal symptoms) (Figure 1f), the effect was not significant, both, clinically, and statistically.

### 3.4 Treatment effects with respect to physical health

**Figure 2:**
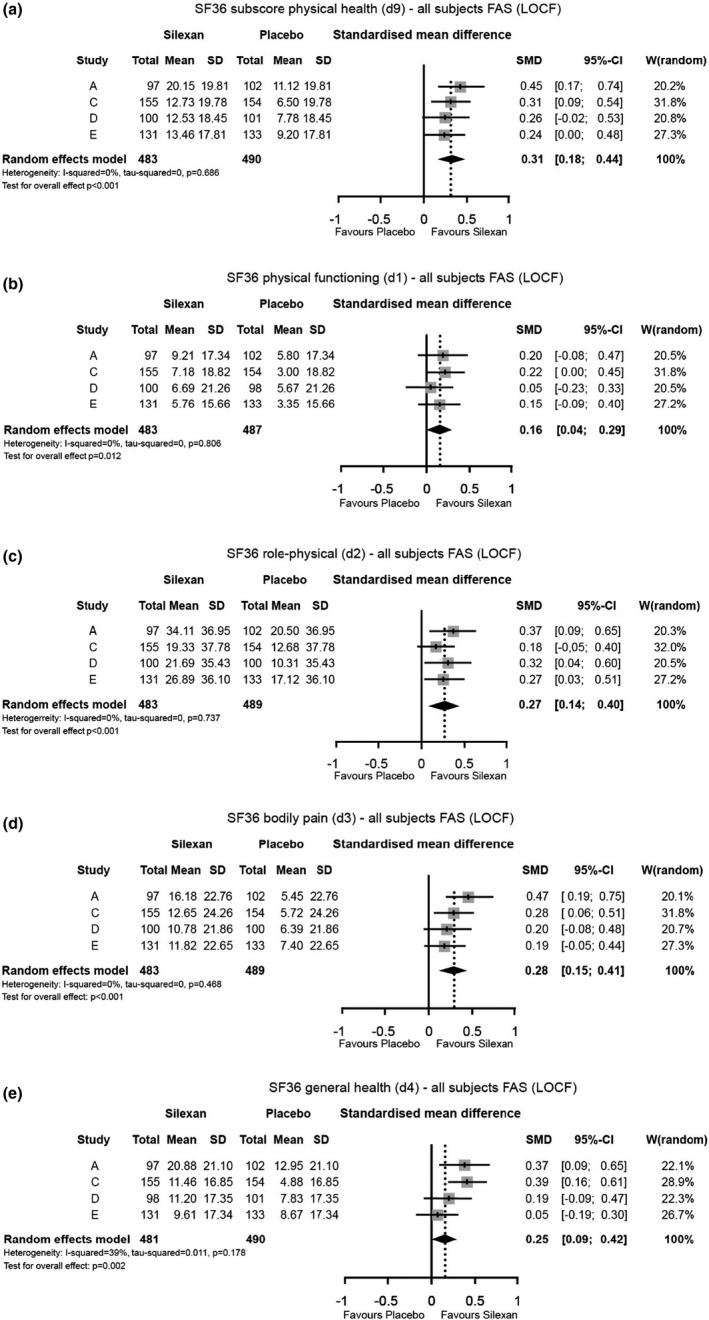
(a–e) Changes of SF‐36 physical health subscore and SF‐36 components describing physical health, between baseline and week 10

In trials A, C, D, and E, physical HRQoL was investigated using the SF‐36 questionnaire. In all 4 single trials, physical health improved more in patients treated with Silexan than in the placebo groups. Treatment group differences were statistically significant in trials A and C (Figure 2a).

Overall, the meta‐analyses revealed statistically significant results with regard to improved physical QoL in favor of Silexan (Figure 2a). Statistically significant and clinically meaningful differences between Silexan 80 mg/day and placebo were also observed for the SF‐36 physical health components role‐physical (Figure 2c), bodily pain (Figure 2d), and general health (Figure 2e). The SMDs of these components ranged between 0.25 (component general health) and 0.31 (subscore physical health), indicating small to medium effects of Silexan. Although a statistically significant difference between Silexan and placebo emerged also for the SF36 component physical functioning (Figure 2b), this effect was of negligible size in clinical terms.

### 3.5 Treatment effects with respect to insomnia complaints and fatigue

**Figure 3:**
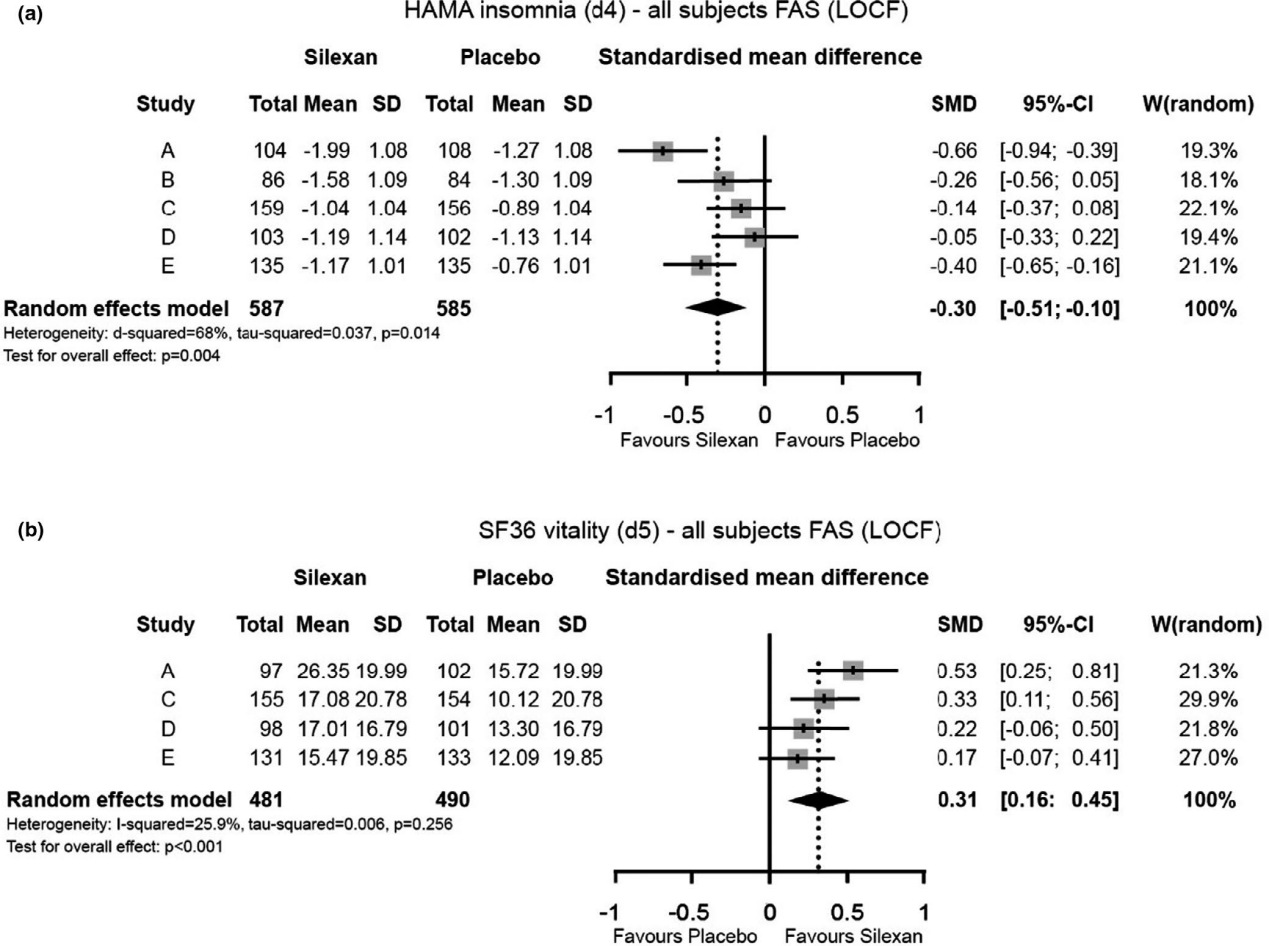
(a and b) Changes of (a) insomnia (HAMA item 4) and (b) fatigue (SF36 vitality), between baseline and week 10

In all 5 single trials, insomnia (HAMA item 4) improved more in patients treated with Silexan than in the placebo groups. The meta‐analysis revealed a statistically significant and clinically meaningful result in favor of Silexan with an effect size of 0.30 (Figure 3a). A similar clinically meaningful difference between Silexan and placebo with a small to medium effect was observed for improved fatigue (Figure 3b).

### 3.6 Treatment effects related to somatic symptoms by gender

In 4 clinical trials (A, B, C, E), treatment effects of Silexan were more pronounced in women as well as in men treated with Silexan compared to women and men of the placebo groups (data not shown). In the bigger subgroup of women, the treatment effect with respect to changes of the HAMA subscore somatic anxiety was statistically meaningful while the difference observed for the smaller subgroup of men showed a similar extent but was not statistically meaningful. This reflects limited statistical power in the smaller subgroup of male patients included, as treatment effects were not significantly different between men and women (*p* = .87) (data not shown).

### 3.7 Treatment effects related to somatic symptoms by age

In total, 998 patients aged < 60 years (502 treated with Silexan 80 mg/day and 496 having received placebo) and 174 patients aged ≥ 60 years (85 treated with Silexan 80 mg/day and 89 having received placebo) were analyzed to describe treatment effects on somatic anxiety (FAS). In 4 clinical trials (A, B, C, E), treatment effects of Silexan were more pronounced in patients of both age groups treated with Silexan compared to both age groups in the placebo groups (subgroup difference *p* = .4764; data not shown). The treatment effect with respect to changes of the HAMA subscore somatic anxiety was statistically meaningful for patients in both age groups (effect size 0.32; *p* < .0001).

## 4 DISCUSSION

The therapeutic effects of Silexan treatment on somatic symptoms, including insomnia complaints and fatigue, and on reduced physical health in patients with anxiety disorders were investigated.

Our results demonstrate that Silexan is superior to placebo in reducing somatic symptoms in patients suffering from at least mild‐to‐moderate subthreshold anxiety disorders or GAD. A statistically significant and clinically meaningful difference in favor of Silexan was shown for the HAMA subscore somatic anxiety. Notably, cardiovascular and genitourinary symptom improvements contributed most to the overall effect of Silexan in reducing somatic anxiety. Among patients with cardiovascular disease, anxiety disorders are common and associated with poor cardiovascular health, including the development and progression of coronary artery disease and heart failure (Celano et al., 2016; Roest et al., 2010). In patients with stable coronary artery disease, two‐year follow‐up data revealed that patients with comorbid GAD had a twofold increased risk of major adverse cardiac events compared to those without GAD (Frasure‐Smith & Lesperance, 2008). The mechanisms mediating the underlying association between anxiety disorders and cardiac disease are poorly understood, although behavioral and physiologic factors have been proposed (Celano et al., 2016). Another important public health concern is the fact that anxiety disorders are common in patients with noncardiac chest pain (Celano et al., 2016; Ortiz‐Garrido et al., 2015). Such patients tend to have similar levels of anxiety and low QoL as patients diagnosed with chest pain of cardiac origin (Webster et al., 2012). Patients with cardiovascular disease are likely to be taking other drugs, notably antihypertensives, lipid‐lowering drugs and antiarrhythmic drugs; hence, prescribing anti‐anxiety agents such as SSRIs is of some concern because of possible pharmacokinetic interactions (Davies et al., 2004). We have shown that Silexan exerts a clinically relevant small to moderate effect on cardiovascular symptoms and therefore is a well‐tolerated alternative to treatment with SSRIs in GAD.

Evidence from the literature suggests that anxiety and genitourinary symptoms might share biological pathways, for example, the serotonergic pathway. Indeed, the SNRI duloxetine has been shown to improve symptoms of overactive bladder in women (Steers et al., 2007). Interestingly, in our study, treatment for 10 weeks with Silexan also improved genitourinary symptoms in patients with anxiety.

Impairment in HRQoL has been noted in patients with anxiety disorders (Beard et al., 2010). Our results show that Silexan is efficacious in improving physical HRQoL in patients with anxiety disorder. The greatest improvements were observed for the SF‐36 physical health components role‐physical and bodily pain. The latter finding is of particular interest as anxiety shares the same pathophysiological pathways as pain with mutual effects on each other (de Heer et al., 2014; Means‐Christensen et al., 2008). Both anxiety disorders and pain facilitate the central modulation of the pain response at multiple sites in the brain (e.g., periaqueductal gray, amygdala, and hypothalamus) (Ossipov et al., 2010). They also share underlying cognitive and behavioral processes, such as increased attention toward threat and anxious avoidance of physical exertion (Asmundson & Katz, 2009; Sareen et al., 2005). Furthermore, anxiety‐induced stress increases the production of pro‐inflammatory cytokines (Hou et al., 2017), which may also increase pain (de Oliveira et al., 2011). In a study by de Heer et al. (2014), anxiety symptom severity was associated with more disabling and severely limiting pain (de Heer et al., 2014). This study also showed that anxiety disorders have a similar and strong association with musculoskeletal pain, cardio‐respiratory pain, and gastrointestinal pain compared to a control group without depressive or anxiety disorder (de Heer et al., 2014).

Silexan does not share the same mechanism of action as other antidepressant or anxiolytic drugs (e.g., benzodiazepines). This may explain why particular side effects typical of benzodiazepines, such as sedation, are absent (Schuwald et al., 2013). The SNRIs duloxetine and venlafaxine are used to achieve reduction in chronic pain associated with depression (Jann & Slade, 2007). It is unclear whether the mechanisms through which Silexan treatment improves somatic symptoms and physical health in anxiety are similar to those attributed to dual‐action antidepressants. However, a study by Baldinger et al. (2014) reported that effects on the serotonin‐1A receptor may contribute to the therapeutic action of Silexan (Baldinger et al., 2014). Alternatively, it is plausible that Silexan reduces anxious arousal thus decreasing patient sensitivity to anxiety and perception of somatic symptoms, including pain. Indeed, some individuals with elevated anxiety sensitivity may be more likely to perceive somatic sensations associated with anxiety as dangerous, which can lead to a perpetuated cycle of increased perception to and misinterpretation of bodily cues (Reiss, 1991), thereby increasing healthcare‐seeking behavior.

Insomnia, commonly accompanied by fatigue, is highly prevalent in anxiety disorders and may also contribute to the maintenance and/or exacerbation of anxiety through its impact on anticipatory brain function (Cox & Olatunji, 2016). Insomnia (according to the HAMA insomnia item) has been reported to be the most frequent complaint among patients presenting with GAD in primary care, with an incidence of 32.5% (Wittchen et al., 2002). In our study, there was an overall improvement in insomnia and fatigue in patients treated with Silexan; this is a secondary effect resulting from anxiolysis (Seifritz et al., 2019). Better sleep may also lead to reduced hyperarousal and perception of pain and/or somatic symptoms associated with anxiety as well as a decrease in fatigue.

In general, women report more bodily distress in terms of more numerous, more intense, and more frequent somatic symptoms than men (Barsky et al., 2001). The fact that the trials analyzed in our meta‐analysis recruited significantly more women than men supports this finding. There was no significant difference in the effect of Silexan treatment between male and female patients included in our analysis, suggesting that Silexan is of clinical benefit for somatic anxiety irrespective of gender.

In addition to gender, other socio‐demographic factors such as age have been identified as correlates of insomnia (Ohayon, 2002; Wennberg et al., 2013). From our data the effects of Silexan on somatic complaints are found independent of age, that is, age did not influence the variance of the results. Evidence from two studies with Silexan in subthreshold anxiety patients with a wide range of ages, including elderly patients > 65 years, support our findings (Kasper, 2015; Seifritz et al., 2019), and however, further randomized studies will be required to confirm this.

Another limitation of this meta‐analysis is that the studies found and included were published by the same research group, which also overlaps with the authors of this work. Therefore, effect sizes could potentially be more similar than effect sizes from studies of different research groups. This is because the effect sizes might be influenced by, for example, the way the variables were analyzed, the subjects were sampled, and the observers or interviewers who collected the data (Cooper, 2009; Van den Noortgate et al., 2013). This is indeed the case, as shown in a similar network meta‐analysis which included 4 papers by Kasper et al. were included in the meta‐analysis by et al.(Yap et al., 2019). Moreover, the studies were carried out according to classical spects, that is Good Clinical Practice, and therefore, the data should be considered robust. Indeed, there are statistically significant and clinically meaningful results in the present study concerning the beneficial effects of Silexan on somatic symptoms and physical health. Future trials may confirm our findings not only in larger samples but also in terms of a sustained effect on somatic anxiety and improved physical health of Silexan. In this context, further research conducted by different research groups is encouraged.

## 5 CONCLUSIONS

In conclusion, this meta‐analysis in patients with anxiety reports statistically significant and clinically meaningful advantages of 80 mg Silexan compared to placebo in improving somatic symptoms, including pain and insomnia, and physical health. This study adds to the empirical evidence supporting a role for the lavender oil preparation Silexan in the treatment armamentarium for anxiety disorders, including those presenting with somatic symptoms.

## Conflict of Interest

Prof. Dr Roland von Känel has received honoraria from Schwabe and Vifor Pharma Switzerland. Prof. Dr Siegfried Kasper has received grants/research support, consulting fees and/or honoraria within the last three years from Angelini, AOP Orphan Pharmaceuticals AG, Celgene GmbH, Eli Lilly, Janssen‐Cilag Pharma GmbH, KRKA‐Pharma, Lundbeck A/S, Mundipharma, Neuraxpharm, Pfizer, Sage, Sanofi, Schwabe, Servier, Shire, Sumitomo Dainippon Pharma Co. Ltd., Sun Pharmaceutical Industries Ltd. and Takeda. Dr Guido Bondolfi has received travel grants and/or honoraria from Schwabe, Servier, Mepha Schweiz AG and Sandoz Pharmaceuticals. He has also participated in advisory boards by Lundbeck and Servier. Prof. Dr Edith Holsboer‐Trachsler has received an honorarium from Schwabe. Dr Josef Hättenschwiler has received travel grants and/or honoraria from Schwabe and Lundbeck. Dr Christian Imboden has received travel grants and/or honoraria from Schwabe, Vifor Pharma Switzerland and Servier as well as taken part in advisory boards organized by Lundbeck and Janssen‐Cilag AG. Prof. Martin Hatzinger has received honoraria from Schwabe. Dr Ellen Heitlinger has received honoraria from Schwabe. Prof. Erich Seifritz has received honoraria from Schwabe GmbH for educational lectures. He has further received educational grants and consulting fees from Janssen Cilag, Lundbeck, Angelini, Otsuka, Servier, Ricordati, Vifor, Sunovion, and Mepha.

## Author Contributions

R.v.K., S.K., G.B., E.H‐T., J.H., M.H., C.I., E.H., and E.S. contributed substantially to the conception and design of this study. They also contributed to the analysis and interpretation of data. R.v.K. and E.H. contributed to the drafting of the manuscript. R.v.K., S.K., G.B., E.H‐T., J.H., M.H., C.I., E.H., and E.S. critically revised the manuscript and gave their final approval for submission.

## ETHICAL STATEMENT

There are no ethical concerns with regard to this meta‐analysis as only data from other randomized trials were re‐analyzed.

### PEER REVIEW

The peer review history for this article is available at https://publons.com/publon/10.1002/brb3.1997.

## Data Availability

Data sharing is not applicable to this article as no new data were created or analyzed in this study.

## Appendix 1

**Table A1:** Baseline scores of HAMA (somatic anxiety subscore, somatic symptoms, and insomnia)

			HAMA																	
			Subscore Somatic anxiety		Item 7 Somatic muscular		Item 8 Somatic sensory		Item 9 Cardiovascular symptoms		Item 10 Respiratory symptoms		Item 11 Gastrointestinal symptoms		Item 12 Genitourinary symptoms		Item 13 Autonomic symptoms		Item 4 Insomnia	
Clinical trial	Treatment groups	*N* ^*^	Mean	*SD*	Mean	*SD*	Mean	*SD*	Mean	*SD*	Mean	*SD*	Mean	*SD*	Mean	*SD*	Mean	*SD*	Mean	*SD*
A	Silexan 80 mg	104	11.2	3.9	2.0	1.0	1.6	1.0	1.8	1.1	1.4	1.1	1.4	1.2	1.2	1.1	1.8	0.9	3.2	0.7
	Placebo	108	11.4	3.5	2.0	1.0	1.6	0.9	2.1	1.2	1.5	1.1	1.3	1.1	1.0	1.0	1.8	0.8	3.3	0.7
B	Silexan 80 mg	86	10.6	4.4	2.0	1.2	1.7	1.2	1.2	1.2	0.9	1.0	1.7	1.3	1.3	1.1	1.8	1.0	3.4	0.7
	Placebo	84	11.0	4.2	2.2	1.1	1.8	1.1	1.3	1.0	0.9	1.0	1.6	1.1	1.3	1.0	2.0	1.0	3.5	0.6
C	Silexan 80 mg	159	11.3	3.3	1.8	1.0	2.0	1.0	1.8	1.0	1.2	0.9	1.6	1.0	1.2	1.0	1.8	0.9	2.4	0.8
	Placebo	156	12.0	3.7	2.0	1.1	2.0	1.0	1.9	1.0	1.4	1.0	1.7	1.1	1.3	1.0	1.8	0.9	2.6	1.0
D	Silexan 80 mg	103	11.7	3.6	2.0	1.1	1.9	1.0	2.0	1.0	1.4	1.1	1.4	1.1	1.2	1.1	1.7	0.8	2.3	0.9
	Placebo	102	11.3	3.8	2.0	1.0	1.8	1.0	1.8	1.1	1.4	1.1	1.5	1.0	1.3	1.0	1.6	0.8	2.3	1.0
E	Silexan 80 mg	135	10.6	3.6	1.9	1.0	1.7	1.0	1.6	1.2	1.2	1.2	1.3	0.9	1.1	1.0	1.8	0.9	2.4	1.1
	Placebo	135	10.3	3.4	1.8	1.0	1.6	0.9	1.6	1.2	1.2	1.1	1.3	1.0	1.0	0.9	1.8	0.9	2.5	1.0
Pooled	Silexan 80 mg	587	11.1	3.7	1.9	1.0	1.8	1.0	1.7	1.1	1.3	1.1	1.4	1.1	1.2	1.0	1.8	0.9	2.7	1.0
	Placebo	585	11.1	3.7	2.0	1.0	1.7	1.0	1.8	1.1	1.3	1.1	1.4	1.1	1.2	1.0	1.8	0.9	2.7	1.0

**Table A2:** Baseline scores of SF‐36 components related to physical health, component vitality (fatigue), and subscore physical health

SF−36 component			Physical Functioning		Role‐Physical		Bodily Pain		General Health		Vitality		Subscore Physical Health	
Trial	Treatment groups	*N*	Mean	*SD*	Mean	*SD*	Mean	*SD*	Mean	*SD*	Mean	*SD*	Mean	*SD*
A	Silexan 80 mg	104	75.8	21.8	39.9	41.7	53.4	28.9	37.7	20.1	28.8	16.5	51.7	21.7
	Placebo	108	73.4	22.6	41.9	40.7	57.6	27.1	39.8	20.2	29.5	19.6	53.2	22.1
C	Silexan 80 mg	103	69.0	25.0	49.0	43.0	60.2	30.8	47.1	15.1	34.8	16.2	56.5	21.6
	Placebo	102	68.4	26.6	50.0	41.1	59.5	28.8	44.7	17.7	33.8	17.8	55.4	21.5
D	Silexan 80 mg	135	75.5	25.8	40.7	41.1	60.0	29.3	40.0	16.4	31.6	17.6	54.1	22.1
	Placebo	135	76.6	20.3	39.6	41.0	59.3	31.0	41.4	19.8	32.0	17.1	54.2	22.2
E	Silexan 80 mg	159	68.3	26.2	33.6	38.8	51.6	32.0	39.3	19.9	27.1	18.5	48.2	23.5
	Placebo	156	69.0	25.0	36.5	40.1	50.2	30.4	41.4	19.7	27.9	18.7	49.3	22.7
Pooled	Silexan 80 mg	501	71.9	25.2	40.0	41.1	56.0	30.6	40.7	18.4	30.2	17.6	52.2	22.6
	Placebo	501	71.9	23.8	41.3	40.8	56.1	29.8	41.7	19.5	30.5	18.4	52.7	22.3
